# Biosynthesis and Characterization of AgNPs–Silk/PVA Film for Potential Packaging Application

**DOI:** 10.3390/ma10060667

**Published:** 2017-06-17

**Authors:** Gang Tao, Rui Cai, Yejing Wang, Kai Song, Pengchao Guo, Ping Zhao, Hua Zuo, Huawei He

**Affiliations:** 1 State Key Laboratory of Silkworm Genome Biology, Southwest University, Chongqing 400715, China; taogang@email.swu.edu.cn (G.T.); 18728301293@163.com (K.S.); guopc@swu.edu.cn (P.G.); zhaop@swu.edu.cn (P.Z.); 2 College of Biotechnology, Southwest University, Chongqing 400715, China; cairui0330@email.swu.edu.cn; 3 College of Pharmaceutical Sciences, Southwest University, Chongqing 400715, China; zuohua@swu.edu.cn; 4 Chongqing Engineering and Technology Research Center for Novel Silk Materials, Southwest University, Chongqing 400715, China

**Keywords:** biosynthesis, AgNPs, silk, PVA, packaging application

## Abstract

Bionanocomposite packaging materials have a bright future for a broad range of applications in the food and biomedical industries. Antimicrobial packaging is one of the bionanocomposite packaging materials. Silver nanoparticle (AgNP) is one of the most attractive antimicrobial agents for its broad spectrum of antimicrobial activity against microorganisms. However, the traditional method of preparing AgNPs-functionalized packaging material is cumbersome and not environmentally friendly. To develop an efficient and convenient biosynthesis method to prepare AgNPs-modified bionanocomposite material for packaging applications, we synthesized AgNPs in situ in a silk fibroin solution via the reduction of Ag^+^ by the tyrosine residue of fibroin, and then prepared AgNPs–silk/poly(vinyl alcohol) (PVA) composite film by blending with PVA. AgNPs were synthesized evenly on the surface or embedded in the interior of silk/PVA film. The prepared AgNPs–silk/PVA film exhibited excellent mechanical performance and stability, as well as good antibacterial activity against both Gram-negative and Gram-positive bacteria. AgNPs–silk/PVA film offers more choices to be potentially applied in the active packaging field.

## 1. Introduction

Bionanocomposite packaging materials have a bright future for a broad range of applications in the food and biomedical industries, in addition to innovative active and intelligent food packaging with biofunctional properties. Antimicrobial packaging is a form of active packaging that is usually achieved by the incorporation or immobilization of potent antimicrobial agents into the packaging system [1]. Nowadays, the ever-growing demand for safe and minimally-processed products presents a major challenge for the packaging industry to develop active packaging materials that can maintain the safety and quality of packaged items [2]. To improve the safety and the shelf-life of packaged products, increasing attention has been paid to the design, development and characterization of films, blends, coatings, and nanocomposite formulations with antimicrobial or antioxidant properties [3,4,5,6]. Various organic acids, enzymes, bacteriocins, chelating agents, spice extracts, and essential oils have been directly incorporated into the films to develop antimicrobial packaging [7,8,9,10]. Among the antibacterial materials, nanoparticles or nanocomposite antimicrobial materials have long been exploited for high efficiency in inhibiting the growth of microorganisms due to high surface-to-volume ratio and enhanced surface reactivity [11,12]. Silver nanoparticle (AgNP) is one of the most attractive antibacterial materials for its broad spectrum of antimicrobial activities against foodborne microorganisms. Such nanocomposite materials with profound antimicrobial function have great value in active packaging for shelf life extension and the safety enhancement of packaged products [13,14].

The traditional method of preparing AgNPs-functionalized material is cumbersome and not environmentally friendly. The synthesis and immobilization of AgNPs requires at least two steps. This approach has been gradually abandoned due to the complicated and tedious procedure [15]. Increasing attentions toward sustainable and environmentally-friendly technologies have promoted the development of AgNPs synthesized via green biological systems. In recent years, the biomineralization approach—a combination of biotechnology and nanotechnology—has developed and provided unparalleled opportunities for natural biomaterials [16,17,18]. Natural macromolecules serve as a biotemplate to prepare novel nanomaterials [19,20]. 

Silk fibroin is a natural protein from silkworm silk, and is a common biomacromolecule with unique sequence-specific self-assembly behavior [21]. Over the past decade, silk fibroin has been applied in tissue engineering as degradable surgical sutures and scaffolds [22,23] for its good biocompatibility, controllable biodegradability, and easy fabrication into different forms, such as fibers, films, gels, and three-dimensional scaffolds [24]. Silk fibroin is a good candidate for biomineralization. Previous works have indicated that silk fibroin regulates the morphologies of inorganic nanoparticles during the biomineralization process [25,26]. Silk fibroin contains 18 types of amino acid residues, including some polar amino acids such as tyrosine (Tyr). Tyr endows silk fibroin with electron-donating property. The electron-donating property of the phenolic hydroxyl group of Tyr could directly reduce silver ion to AgNP [27]. Thus, it is possible to synthesize AgNPs through the reduction of Ag^+^ by silk fibroin in situ to prepare the antibacterial silk film.

Biopolymer film such as AgNPs–silk is limited in its packaging application due to its poor mechanical property. To improve the mechanical property, biopolymer–polymer interaction is developed by blending natural biopolymers with polymers. Poly(vinyl alcohol) (PVA) is a biodegradable, biocompatible, water-soluble and non-toxic semi-crystalline polymer. It offers good thermo-mechanical property, thermal stability, mechanical strength and flexibility, as well as good optical and physical properties that are crucial for packaging application [28,29]. Moreover, PVA is approved by the food and drug administration as an indirect food additive for flexible food packaging [30,31]. The combination of AgNPs, silk fibroin, and PVA will be promising for active packaging.

The aim of this work was to prepare a kind of biodegradable film with antimicrobial activity for packaging application. Here, we have developed a green biosynthesis method to prepare AgNPs–silk/PVA film. Fourier transform infrared spectroscopy (FTIR) and field emission scanning electron microscopy (FESEM) were performed to characterize the chemical groups and the surface morphologies of AgNPs–silk/PVA films, respectively. The mechanical and thermal properties of the prepared films were investigated by the stress–strain assay and thermogravimetric analyzer (TGA), respectively. The antimicrobial activities of AgNPs–silk/PVA films are evaluated by the bacterial growth curve and inhibition zone assays. Our studies suggest that the prepared AgNPs–silk/PVA film has great potential in active packaging applications.

## 2. Results and Discussion

### 2.1. Preparation of AgNPs–Silk/PVA Film

**Figure 1:**
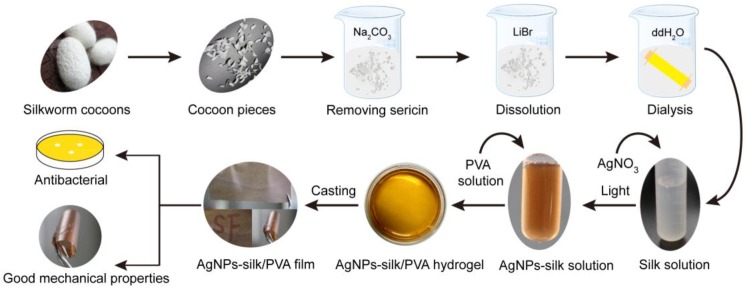
Illustration of the preparation and analysis of silver nanoparticles (AgNPs)–silk/poly(vinyl alcohol) (PVA) film.

AgNPs synthesis via the reduction of Ag^+^ by Tyr residue has been developed [32,33,34]. The aim of this study is to prepare AgNPs–silk film as an antibacterial packaging material. However, the poor mechanical performance of AgNPs–silk film limits its application in packaging material. The mechanical property of a film can be improved via crosslinking, copolymerizing, and blending with other macromolecular materials [35,36]. PVA has good film-forming properties [37]. PVA blending is widely applied in packaging materials for its good mechanical property and excellent barrier property toward hydrocarbons and gases [38,39]. However, bacteria can easily adhere and grow on PVA film, thus resulting in the deformation and the degradation of PVA film. Therefore, it is highly desired to prepare a PVA composite film with broad antimicrobial activity to facilitate its application in packaging material. In this study, we developed a green, facile, and economical approach to prepare AgNPs–silk/PVA film without the addition of any other chemical agents, as illustrated in Figure 1.

### 2.2. Characterization of Silk Fibroin

**Figure 2:**
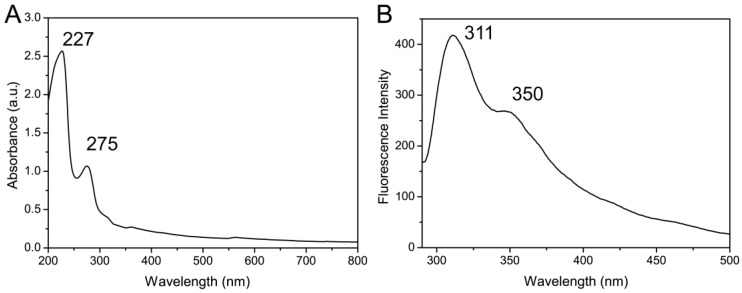
Characterization of silk fibroin solution by (**A**) UV-vis spectra and (**B**) fluorescence spectra.

To determine whether silk fibroin was successfully extracted from silk cocoon, UV-vis spectroscopy was applied to measure the characteristic absorption peaks of peptide bonds and aromatic amino acids. Two peaks at 227 nm and 275 nm were observed in the absorbance spectra of silk fibroin (Figure 2A), which could be attributed to the absorbance of the aromatic amino acids and peptide bonds of silk fibroin, respectively [40]. 

Fluorescence spectroscopy is an efficient method to analyze silk fibroin, as it has inherent fluorescence from the tyrosine, tryptophan, and phenylalanine residues of silk fibroin. The fluorescence emission spectrum showed two peaks at 311 nm and 350 nm (Figure 2B), which may be attributed to the fluorescence from tyrosine and phenylalanine residues, respectively [41]. These results suggested silk fibroin was successfully extracted from silk cocoon.

### 2.3. Stability of AgNPs–Silk Fibroin Solution

**Figure 3:**
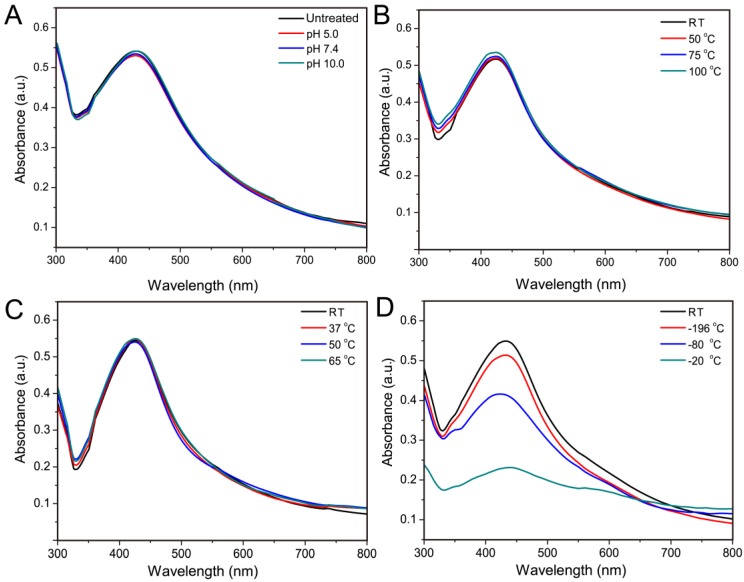
Comparison of UV-vis spectra of AgNPs–silk solutions: (**A**) in pH 5.0, 7.4, and 10.0; (**B**) at room temperature (RT), 50 °C, 75 °C, and 100 °C; (**C**) at room temperature, 37 °C, 50 °C, and 65 °C for 15 days; (**D**) at room temperature, −20 °C, −80 °C, and −196 °C for 15 days.

Individual AgNP is easily oxidized to aggregate in air, which may affect its antibacterial activity; therefore, the stability of AgNP is a critical consideration for its application [42]. In this study, AgNPs–silk solutions were treated at different pHs and temperatures to characterize their stability with UV-vis spectroscopy The results showed the band at 445 nm did not change in either pH 5.0, 7.4, and 10.0 (Figure 3A) or at 50 °C, 75 °C, and 100 °C (pH 5.0) (Figure 3B). When placed at 37 °C, 50 °C, and 65 °C (pH 5.0) for 15 days, the UV absorption spectra of AgNPs–silk did not change (Figure 3C). These results suggested AgNPs–silk solutions had good stability at different temperatures or under different conditions. Silk fibroin could enhance the stability of AgNPs in aqueous dispersions via the interactions with AgNPs. However, under low-temperature conditions, the UV absorption of AgNPs was lower than that at room temperature (Figure 3D). This may be attributed to the aggregation of fibroin under low-temperature conditions.

### 2.4. Characterization of AgNPs–Silk/PVA Film

**Figure 4:**
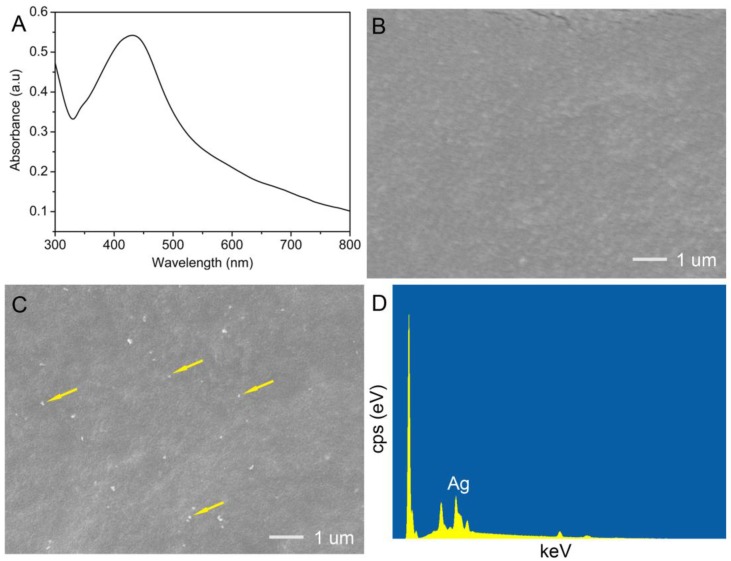
(**A**) UV-vis spectra of AgNPs–silk solution; (**B**,**C**) SEM images of silk/PVA and AgNPs–silk/PVA films; (**D**) EDS of a selected area of AgNPs–silk/PVA film.

To detect the formation of AgNPs, the UV-vis spectrum of AgNPs–silk solution was scanned, as shown in Figure 4A. The result showed a broad absorption band centered at 445 nm, indicating the formation of AgNPs in the solution. The 445 nm absorbance peak may be assigned to the surface plasmon resonance band of AgNP [43]. A typical absorbance peak of AgNP is located around 400 nm. The absorbance peak of AgNPs–silk red-shifted from 400 nm to 445 nm. This may be caused by the high dielectric constant of the peptide matrix, which reduces the plasmon resonance frequency of AgNPs in solution [44].

FESEM images were captured to explore the surface morphologies of silk/PVA and AgNPs- silk/PVA films. Silk/PVA film had smooth surfaces (Figure 4B), implying that the film had a dense structure and good mechanical property. It was noted that some small dots appeared on the surface of the AgNPs–silk/PVA film (Figure 4C), indicating the efficient synthesis of AgNPs in situ. The uniform distribution of AgNPs on the film suggested that silk fibroin was a good dispersant. Aggregations of AgNPs were not observed on the film, and thus it is expected to have a better antimicrobial performance. 

Energy dispersive X-ray spectroscopy (EDS) was carried out to further determine the synthesis of AgNPs in situ on the film. A distinct peak was observed in the EDS spectrum (Figure 4D), which could be assigned to metallic silver. The results suggested that AgNPs were successfully synthesized in situ on the silk/PVA film.

**Figure 5:**
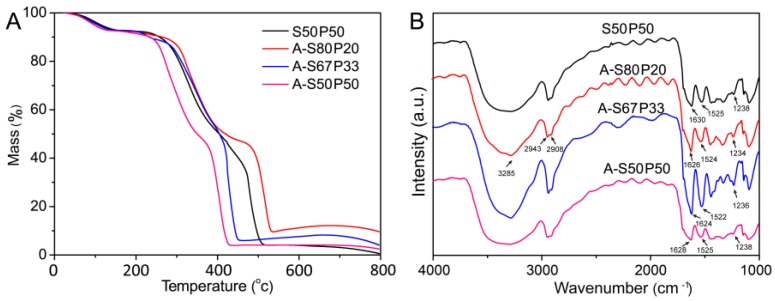
Characterization of silk/PVA and AgNPs–silk/PVA films by (**A**) thermogravimetric analysis (TGA) and (**B**) Fourier transform infrared spectroscopy (FTIR).

The thermal behaviors of silk/PVA and AgNPs–silk/PVA films were analyzed by TGA. The mass of the film as a function of temperature is shown in Figure 5A. The TGA curves of AgNPs–silk/PVA films showed a similar tendency. Compared to silk/PVA film, the residual mass of AgNPs–silk/PVA films were in the range of 3–10%, implying the presence of AgNPs in the silk/PVA film. The mass percentages of AgNPs in A-S80P20, A-S67P33, and A-S50P50 films were calculated to be 10.8%, 6.2%, and 3.6%, respectively, which were consistent with the residual mass percentages of AgNPs–silk/PVA films.

At the first stage, while increasing temperature to 100 °C, water evaporation caused the initial mass reduction of all films. The mass reduction of the film was almost 8%, indicating that the film was a slightly hygroscopic material. At the second stage, while increasing temperature from 200 °C to 330 °C, the mass reduction of the films could be ascribed to the degradation of exposed side chains of silk fibroin or PVA. The dramatic mass reduction occurred in the temperature range of 330–550 °C. This may be attributed to the breakdown of main chain groups of silk fibroin and the decomposition of PVA [45]. At the last stage, silk/PVA film was almost completely lost, whereas AgNPs–silk/PVA films had residual mass ranging from 3% to 10%, indicating the successful synthesis of AgNPs in the silk/PVA film. The A-S50P50 film reached equilibrium at around 420 °C on the TGA curve, which was lower than that of the A-S80P20 film. This result indicated that a high content of silk fibroin could enhance the thermostability of silk/PVA films and delay the thermal degradation process.

It was noted that S50P50 and A-S50P50 showed a plateau at near 3% in a large temperature interval from 450 °C to 700 °C, and only afterwards did S50P50 degrade to zero mass. A-S50P50 had 3% residual mass after 450 °C, which may be ascribed the existence of about 3% AgNPs in the film. The thermal behavior of S50P50 in 450–700 °C may be due to the existence of strong interactions between silk and PVA, which stabilized the S50P50 film and kept 3% residual mass of the film. However, after 700 °C, high temperature destroyed the strong interactions of silk and PVA and resulted in the complete mass loss of S50P50 film.

FTIR spectroscopy was applied to characterize the chemical groups of silk/PVA and AgNPs- silk/PVA films (Figure 5B). All films showed similar peaks. The bands at 3284–3287 cm^−1^ were assigned to the hydroxyl groups of silk and PVA. The bands at 2943 cm^−1^ were attributed to the saturated C–H groups (–CH_3_). The bands at 2908 cm^−1^ were attributed to C–H stretching vibrations (–CH_2_). The amide I (1624–1638 cm^−1^), II (1514–1527 cm^−1^), and III (1232–1250 cm^−1^) bands were believed from silk fibroin in the films [46]. While AgNPs were synthesized in situ on the silk/PVA film, the characteristic peaks such as 3285 cm^−1^, 1624–1630 cm^−1^, 1522–1525 cm^−1^, and 1234–1238 cm^−1^ did not change, suggesting that AgNPs modification did not affect the inherent structure of silk/PVA film.

### 2.5. Mechanical Property of AgNPs–Silk/PVA Film

**Figure 6:**
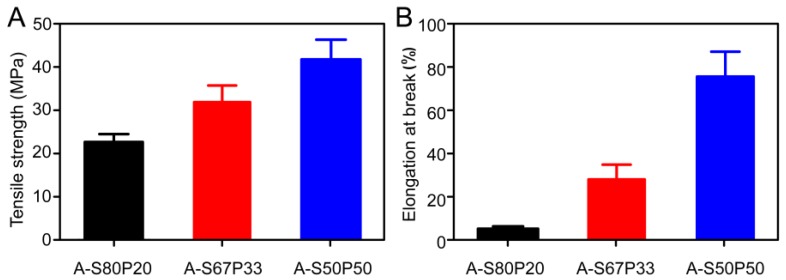
Mechanical properties of AgNPs–silk/PVA film: (**A**) tensile strength and (**B**) elongation at break.

The mechanical properties of AgNPs–silk/PVA films were characterized by their stress–strain curves, as shown in Figure 6. The results showed that with increasing PVA concentration from 20% to 50%, the tensile strength of AgNPs–silk/PVA film increased significantly (Figure 6A). The tensile strength of the A-S50P50 film was 42 ± 6 MPa, which was the highest among all of the tested films. The A-S80P20 film had 5% elongation percentage (Figure 6B), which was the lowest among all of the tested films. With increasing PVA concentration from 20% to 50%, the elongation percentage increased from 5% to 78%, respectively. The elongation percentage of A-S50P50 film was 77 ± 10%, which was the highest among all of the tested films. These results suggested that increasing the PVA concentration could enhance the tensile strength and the elongation at break of AgNPs–silk/PVA film. The A-S50P50 film had the best mechanical performance among all of the tested films, which may be suitable for potential packaging applications. 

### 2.6. Hydrophobicity Measurement

**Figure 7:**
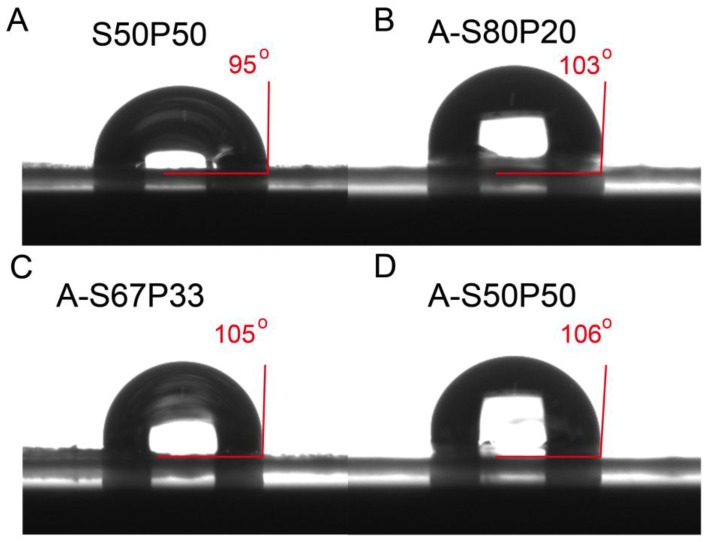
Water contact angles of S50P50 (**A**), A-S80P20 (**B**), A-S67P33 (**C**), and A-S50P50 (**D**) films.

Water contact angle measurements were carried out to determine the hydrophobicity of the silk/PVA film and the AgNPs–silk/PVA film. As shown in Figure 7A, the water contact angle of silk/PVA film was 95°, indicating that it had a relatively hydrophobic surface property. This may be because silk contains some non-polar amino acids. While blending with PVA in different ratios, the water contact angles increased to 103°, 105°, and 106° (Figure 7B–D), indicating AgNPs–silk/PVA film exhibited a certain degree of hydrophobicity. Compared to silk/PVA film, the increase of water contact angles may be due to the presence of AgNPs in the silk/PVA film. Regardless, the resulting water contact angles suggested AgNPs–silk/PVA film had a certain degree of hydrophobicity which may be suitable for packaging applications.

### 2.7. Antibacterial Assay of AgNPs–Silk/PVA Film

**Figure 8:**
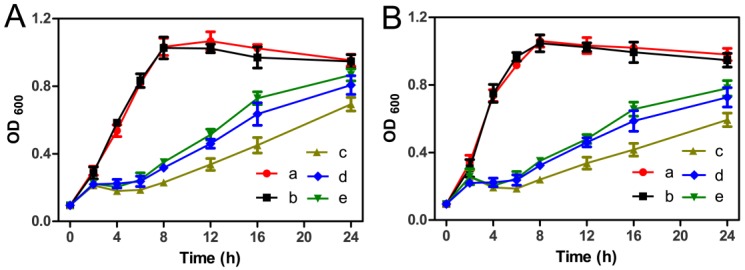
Effects of films on the growth of (**A**) *E. coli* and (**B**) *S. aureus* in the absence of a film (curve a), and in the presence of S50P50, A-S80P20, A-S67P33, and A-S50P50 films (curves b–e).

The antibacterial activity of silk/PVA and AgNPs–silk/PVA films were carefully investigated by the bacterial growth curve assay. *E. coli* and *S. aureus* were selected as the models of Gram-negative and Gram-positive bacteria, respectively [40]. Silk/PVA film itself did not affect the growth of bacteria (Figure 8, curve b). However, AgNPs–silk/PVA films significantly inhibited the growth of bacteria. In the presence of AgNPs–silk/PVA films, the lag phase of both *E. coli* and *S. aureus* lasted from less than 1 h to about 6 h (Figure 8, curves c–e). Even at the exponential phase, the growth rate of bacteria in the presence of AgNPs–silk/PVA film was not as fast as that of control (Figure 8, curves a,b), indicating that the prepared AgNPs–silk/PVA films had good antibacterial activity. 

In this study, we prepared the AgNPs–silk/PVA films with different ratios of AgNPs–silk and PVA (4:1, 4:2, and 4:4). Thus, the AgNPs contents were different in these prepared AgNPs–silk/PVA films, which resulted in the difference of the antibacterial ability of AgNPs–silk/PVA films (Figure 8). A-S67P33 and A-S50P50 showed almost the same tendency from 0 h to 8 h. However, A-S67P33 and A-S50P50 showed small differences in the antibacterial ability for the difference in the AgNPs contents after 8 h. As A-S80P20 had greater AgNPs contents than A-S67P33 and A-S50P50, and A-S80P20 showed a more obvious inhibitory effect on bacterial growth than A-S67P33 and A-S50P50.

The FESEM results showed that AgNPs were evenly dispersed on the surface of silk/PVA film. In addition, the synthesized AgNPs were wrapped in the interior of silk/PVA film. During the bacterial growth, AgNPs exposed on the surface of film were gradually released into the media to inhibit bacterial growth, then AgNPs embedded in the interior of the film were released into the media to continuously inhibit the growth of bacteria.

**Figure 9:**
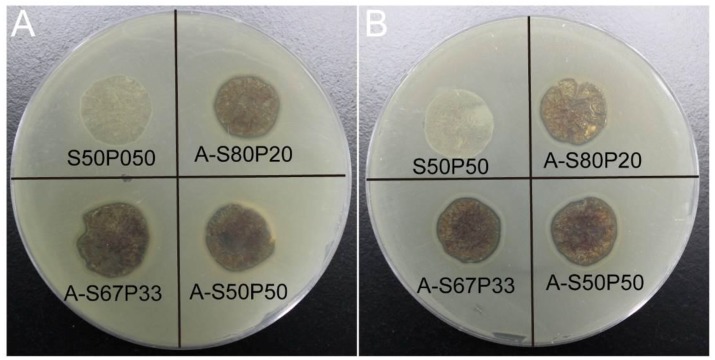
Inhibition zone assay of silk/PVA and AgNPs–silk/PVA films on the growth of (**A**) *E. coli* and (**B**) *S. aureus*.

An inhibition zone assay was performed to further evaluate the antibacterial ability of AgNPs- silk/PVA film, as shown in Figure 9. The results showed that silk/PVA film did not affect the growth of *E. coli* and *S. aureus*, which was consistent with the observation of the growth curve assay. However, AgNPs–silk/PVA films inhibited the growth of bacteria both for *E. coli* and *S. aureus*, as observed by the formation of obvious inhibition zones (Figure 9). These results suggested that AgNPs–silk/PVA film could effectively inhibit the growth of both Gram-negative and Gram-positive bacteria.

### 2.8. Degradation of AgNPs–Silk/PVA Film

**Figure 10:**
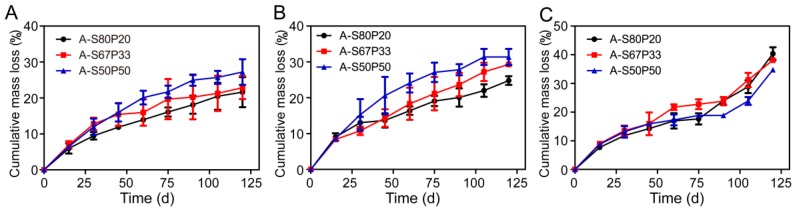
Degradation kinetics of AgNPs–silk/PVA films in (**A**) pH 4.0, (**B**) 7.4, and (**C**) 10.0 buffers at 37 °C.

The stability of a film is directly related to its performance. Additionally, the controllable degradation of antibacterial film is essential for the delayed release of AgNPs to maintain sustained antibacterial activity. To evaluate the degradation of AgNPs–silk/PVA film in the real world, the mass reduction resulting from the degradation of AgNPs–silk/PVA film was examined in PBS buffers with different pHs. After 120 days of treatment in pH 4.0 buffer, AgNPs–silk/PVA film still maintained a good shape. The mass reduction of AgNPs–silk/PVA film was less than 30% (Figure 10A). In contrast, in pH 7.4 and 10.0 buffers, the shape of the films had been changed after 120 days of treatment. The mass reduction was close to 30% in pH 7.4 buffer or 40% in pH 10.0 buffer (Figure 10B,C). 

Silk has an isoelectric point of 4.6. In addition, PVA shows weak acidity in water. Thus, under acidic conditions, AgNPs–silk/PVA film had a relatively better stability. The degradation of AgNPs–silk/PVA film in a neutral environment was faster than that in an acidic environment. Of note, the degradation behavior of all films was similar under acidic or neutral conditions—the content of silk fibroin affected the degradation rate. The higher the silk fibroin content, the slower the degradation rate. However, under alkaline conditions, the lower the silk fibroin content, the slower the degradation rate. These results suggested that the prepared AgNPs–silk/PVA films had good stability under different conditions.

## 3. Materials and Methods

### 3.1. Materials

Silkworm cocoons were kindly provided by the State Key Laboratory of Silkworm Genome Biology, Southwest University, China. Poly(vinyl alcohol) (PVA) and silver nitrate (AgNO_3_) (AR, 99.99%) were purchased from Aladdin Corp. (Shanghai, China). Mili-Q water was prepared by a water purification system (Millipore, Billerica, MA, USA) and used in all experiments.

### 3.2. Preparation of Regenerated Silk Fibroin Solution

Silk cocoon consists of fibroin and sericin, wherein fibroin is wrapped by sericin. Degumming and dissolving of silk fibroin followed the established procedures. In brief, silk fibers were degummed twice with 0.5% NaHCO_3_ (wt %) solution at 100 °C for 30 min, then washed with water twice and air-dried at room temperature. The degummed silk fibers were dissolved in 9.3 mol/L LiBr solution, and then dialyzed against water for 48 h at room temperature with a semipermeable membrane (MEMBRA-CEL, 12,000–14,000 MWCO) to remove LiBr. Silk fibroin solution was centrifuged after dialysis. Then, the supernatant was collected and stored at 4 °C. 

### 3.3. Synthesis of AgNPs 

AgNO_3_ powders (400 mg) were added to 100 mL of 2% silk solution (wt %). The final AgNO_3_ concentration was 4 mg/mL. Then, the AgNO_3_–silk solution was irradiated with natural light at room temperature for 24 h to prepare AgNPs–silk solution.

### 3.4. Preparation of AgNPs–Silk/PVA Film

PVA powder was dissolved in water to a final concentration of 2% (wt %) under a constant stirring speed of 200 rpm/min at 85 °C until it was completely dissolved. AgNPs–silk solution (2%) and PVA solution (2%) were mixed thoroughly as the volume ratios of 4:1, 4:2, and 4:4. The mixed solution was placed onto the petri-plates (diameter = 90 mm), and then frozen under −20 °C and thawed at room temperature for four cycles to form AgNPs–silk/PVA hydrogel. The hydrogel was further dried at 65 °C to form AgNPs–silk/PVA film, which were termed as A-S80P20, A-S67P33, and A-S50P50, respectively.

### 3.5. Characterization of AgNPs–Silk/PVA Film

The surface structure and morphology of AgNPs–silk/PVA films were studied on a field emission-SEM of JSM-7800F (JEOL, Tokyo, Japan). EDS spectra were measured by INCA X-Max 250 (Oxford Instruments, Concord, MA, USA) during SEM tests for the analysis of chemical elements. All samples were sputter-coated with platinum for 90 s before observation. The FTIR spectrum was collected in the range of 400–4000 cm^−1^ on a Nicolet iz10 IR microscope (Thermo Fisher Scientific, Waltham, MA, USA). UV-vis spectra were measured on a DU-800 UV-vis spectrophotometer (Beckman Coulter, Brea, CA, USA) in a 1 cm light path cuvette from 300 nm to 800 nm. Fluorescence emission spectra were recorded at room temperature on a Hitachi F-7000 fluorescence spectrophotometer (Hitachi, Tokyo, Japan). The excitation wavelength was 280 nm and the detection wavelength range was 290–500 nm. Thermal stability was measured on a TGA-Q50 thermogravimetric analyzer (TA instruments, New Castle, DE, USA). The heating rate was 10 °C/min in N_2_ with the flow rate of 20 mL/min. The tensile strength and the elongation at break of the films were determined according to ASTM standard method on an AG-X plus universal testing machine (Shimadzu, Kyoto, Japan). The films were cut into rectangular shapes with a dimension of 1 cm × 3 cm (length × width) and then used for measurement.

### 3.6. Bacterial Growth Curve Assay

Bacterial growth curve assay was performed to evaluate the antibacterial activity of AgNPs–silk/PVA film according to a previous method [47]. Bacteria in lag phase were inoculated into 10 mL Luria-Bertani (LB) medium, and cultured at 37 °C in the presence of silk/PVA or AgNPs–silk/PVA films. Bacterial suspensions (0.5 mL) were collected at different intervals to measure the optical density at the wavelength of 600 nm (OD_600_). All growth curve tests were made in triplicate to ensure that the results were repeatable.

### 3.7. Inhibition Zone Assay

The antimicrobial activity of AgNPs–silk/PVA film against *E. coli* and *S. aureus* was evaluated by an inhibition zone assay as a previous report [48]. About 100 μL of bacterial suspension (1 × 10^8^ CFU/mL) was spread on LB agar plate, then the circular film with a diameter of 1 cm was placed on the surface of agar plate. After 12 h incubation at 37 °C, the diameters of inhibition zones were measured to evaluate the antibacterial activities of the films.

### 3.8. In Vitro Degradation Analysis

AgNPs–silk/PVA films (2 cm × 2 cm) were immersed in pH 4.0, 7.4 and 10.0 buffers at 37 °C. PBS buffer was replaced daily. At given time points, the films were taken out from PBS buffers, washed, dried, and weighed. The degradation was calculated as the difference between the initial and dry masses at given time points divided by the initial dry mass. All tests were performed in triplicate.

## 4. Conclusions

In this study, we have developed an efficient and convenient biosynthesis method to prepare AgNPs–silk/PVA films using natural silk as the reducing and stabilizing agent. AgNPs are synthesized evenly on the surface or embedded in the interior of silk/PVA film. The prepared AgNPs–silk/PVA film has good mechanical performance and stability, as well as good antibacterial activity against both Gram-negative and Gram-positive bacteria, which offers more choices for potential applications in the active packaging field.
